# Accelerated dl-Amino Acid Quantification of Deep Sea Water from Toyama Bay and Anti-Aging Activity Investigation Using *Caenorhabditis elegans*

**DOI:** 10.1021/acs.analchem.5c07605

**Published:** 2026-03-11

**Authors:** Takahiro Takayama, Haruto Iwata, Reina Fujio, Ayaka Minamida, Yuko Sakaguchi, Koichi Inoue

**Affiliations:** † Laboratory of Clinical & Analytical Chemistry, College of Pharmaceutical Sciences, 197547 Ritsumeikan University, 1-1-1 Nojihi-gashi, Kusatsu, Shiga 525-8577, Japan; ‡ College of Pharmaceutical Sciences, Ritsumeikan University, 1-1-1 Nojihi-gashi, Kusatsu, Shiga 525-8577, Japan

## Abstract

Deep seawater (DSW), defined as seawater deeper than 200 m, has notable applications in various fields such as energy, agriculture, food, cosmetics, and public health. Several studies have attributed its utility to mineral effects; however, its organic compounds have rarely been investigated. To support mechanistic evidence related to DSW, a sensitive analytical method was developed for the individual analysis of d- and l-amino acids (AAs) using enantiochemical tagging–liquid chromatography–tandem mass spectrometry. A novel reagent, 2,4-dichloro-6-methoxy-1,3,5-triazine-d-leucine, was developed to enable high-speed analysis of individual dl-AAs, allowing quantification of 19 proteogenic and 2 nonproteogenic dl-AAs within 17 min. Furthermore, this reagent facilitated rapid tagging of target AAs within 10 min of reaction. A limit of detection of 10–100 pmol/L (in vial) was achieved, which was sufficient to characterize the dl-AA profiles in DSW. Three batches of DSW from Toyama Bay were quantitatively analyzed using the spiking standard method, revealing dl-AA concentrations of 10–100 nmol/L. Notably, d-leucine, d-valine, d-alanine, d-serine, d-threonine, and d-glutamic acid were detected at higher concentrations than other d-AAs. A lifespan assay using the *Caenorhabditis elegans* model demonstrated that DSW exhibited a clear proliferative effect, similar to the positive control. Moreover, dl-AA analysis revealed a reduction in d-aspartic acid, an established aging marker, in the proliferated groups.

## Graphical abstract

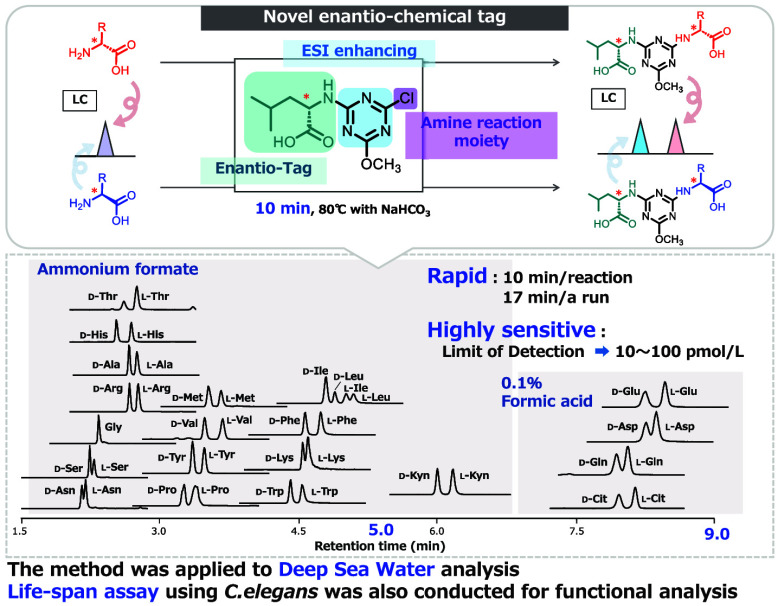

In Japan, deep seawater (DSW) is defined as water deeper than 200 m below the sea surface. DSW is characterized by high purity, stability at low temperatures, high mineral concentrations, and the presence of bioactive nutritional species.^1^ Consequently, its applications have expanded into the fields of energy, agriculture, food, cosmetics, and public health.^2^ In human healthcare, several clinical trials have reported beneficial effects on hypercholesterolemia, allergy, and *Helicobacter pylori* infection.^3–5^ Because the efficacy of DSW has only been partially established, mechanistic studies have attempted to identify which components contribute to its effects. Several studies have reported the relative efficacy of DSW in maintaining mineral balance.^6–8^ For example, Lee et al. demonstrated that the hardness of DSW, particularly the Mg and Ca content ratio (Mg/Ca = 3/1), is critical for reducing hepatic cholesterol production.^6^ Fukui et al. analyzed the hardness of an adjusted solution containing DSW in relation to obesity in mice fed a high-fat diet.^7^ Notably, they analyzed the mineral composition of DSW using liquid chromatography–inductively coupled plasma mass spectrometry (LC–ICP–MS) and found that high levels of Mg and Ca may play a key role in its efficacy. Although reasonable explanations have been proposed regarding the role of minerals in DSW, few studies have focused on amino acids (AAs).

The contribution of AAs in DSW remains largely unexplored. For example, a previous study detected 17 types of AAs in DSW from the Gulf of Mexico, suggesting that organic compounds may contribute to its efficacy.^9^ Ikari et al. analyzed the relationship between the kynurenine (Kyn) signal in DSW from Japan and the stress response in *Paralichthys olivaceus*.^10^ As expected, these studies reported extremely high amounts of minerals such as Mg in DSW, with AAs partially contributing as effective factors. However, analytical data on AAs in DSW remain limited.

AAs are generally proteinogenic l-AAs (19 types), with glycine (Gly) as the achiral exception. Both d- and l-isomers exist, with l-forms typically present in high amounts in biological specimens. Several studies have detected d-AAs in biological samples and reported diverse effects on living organisms.^11–13^ For example, d-serine (Ser) acts as a neurotransmitter for the *N*-methyl-d-aspartate receptor,^12^ while d-alanine (Ala) serves as a precursor for neurotransmitters and osmotic compounds in marine organisms such as shrimp and crabs.^13^ In addition, recent biological research has highlighted the utility of d-AAs, including d-Ala and d-Ser. d-AAs are produced and used by microorganisms such as bacteria and plankton.^14,15^ Notably, one study suggested that d-AAs are selectively used by deep-sea microorganisms,^14^ which may indicate the presence and role of d-AAs in living organisms in DSW. However, analytical research on AAs in DSW remains limited, and even fewer studies on d-AAs have been conducted. Furthermore, because DSW is highly dilute, a sensitive and selective method is required to analyze dl-AAs in DSW.

dl-AAs cannot be analyzed using conventional liquid chromatography–tandem mass spectrometry (LC–MS/MS) because enantiomers exhibit identical physical properties. However, several methods employing chiral column technology or precolumn chiral derivatization have been reported. Chiral derivatization enables enantioseparation in conventional LC mode and enhances MS detection sensitivity by improving ionization efficiency.^16–18^ Several methods employing innovative derivatization reagents have been successfully used to analyze dl-AAs in various biological samples, including our own methodologies.^19–21^ Although these methods achieve rapid analysis (∼20 min/measurement) of proteinogenic AAs with enantioseparation, higher-throughput approaches are required to meet the demands of large-scale data generation. In this study, we screened several novel reagents and developed methods that are more rapid than those used in previous studies, enabling analysis of dl-AAs, including nonproteinogenic AAs such as citrulline (Cit) and Kyn. In addition, DSW from Toyama Bay, Japan was analyzed to profile dl-AA concentrations.

Furthermore, the effectiveness of DSW was evaluated using *Caenorhabditis elegans* in a lifespan assay. The nematode *C. elegans* is approximately 1 mm in length (∼1000 cells in the body) and has a transparent body, which facilitates the observation of organ tissues. It possesses muscles, a digestive tract, a nervous system, epithelium, and reproductive organs. Moreover, its short lifespan of approximately 20 d reduces generation time, making it suitable for lifespan assays.^22–24^ The life extension performance of DSW was assessed using *C. elegans*, and dl-AAs in the bodies were analyzed using the developed method.

## Experimental Section

### Reagents and Chemicals

dl-AA standards, Gly (not optically active), Ala, Ser, threonine (Thr), proline (Pro), valine (Val), leucine (Leu), isoleucine (Ile), aspartic acid (Asp), asparagine (Asn), glutamic acid (Glu), glutamine (Gln), methionine (Met), phenylalanine (Phe), tyrosine (Tyr), tryptophan (Trp), histidine (His), arginine (Arg), lysine (Lys), Cit, and Kyn, were purchased from Tokyo Chemical Industry (Tokyo, Japan). The dl-AA optical purities, expressed as enantiomeric excess (ee, %), as reported in the certificate of analysis, are listed in Table S1. Working solutions of each dl-AA were prepared in acetonitrile/water (1:1) at a concentration of 10 mmol/L. In addition, 2,4-dichloro-6-methoxy-1,3,5-triazine (DCMT) was also purchased from Tokyo Chemical Industry for reagent synthesis. Acetonitrile (CH_3_CN), methanol (CH_3_OH), ethyl acetate (AcOEt), *n*-hexane (Hex), pyridine, triethylamine (TEA), hydrochloric acid (HCl), sodium bicarbonate (NaHCO_3_), ammonium formate, sodium chloride (NaCl), and LC–MS grade formic acid (FA) were obtained from Fujifilm Wako Pure Chemical Co. (Osaka, Japan). Purified water was supplied by a Milli-Q eq 7000 system (Merck, Darmstadt, Germany).

### Screening of the Ideal Enantiochemical Tagging Reagent for dl-AA Analysis

DCMT powder (360 mg, 2.0 mmol) was dissolved in 20 mL CH_3_CN. In a separate flask, L-AAs (l-Phe, l-Pro, l-Val, or l-Leu; each 2.0 mmol) were dissolved in 20 mL of a mixture of H_2_O and CH_3_OH (1:4 v/v). These solutions were mixed for 3 h at room temperature, with 35 μL of TEA added every 30 min. After the reaction was completed, the mixture was extracted into the aqueous layer of a water/Hex phase, and a small amount of HCl was added. Subsequently, the mixture was extracted with AcOEt and Hex (1:1 v/v), washed with H_2_O and NaCl-saturated solutions, and evaporated under reduced pressure. The resulting residues were purified by preparative thin-layer chromatography (pTLC) (silica gel 70 F_254_ PLC, 1.0 mm, Fujifilm Wako Pure Chemical Co.) using an eluent of AcOEt and Hex (1:1 v/v).

### Synthesis of Reagent

DCMT powder (360 mg, 2.0 mmol) was dissolved in 20 mL of CH_3_CN. In a separate flask, d-Leu (262 mg, 2.0 mmol) was dissolved in 20 mL of an H_2_O/CH_3_OH mixture (1:4 v/v). These solutions were mixed for 3 h at room temperature, with 35 μL of TEA added every 30 min. Following completion of the reaction, the mixture was extracted into the aqueous layer of a water/Hex phase, and a small amount of HCl was added. Subsequently, the mixture was extracted with AcOEt and Hex (1:1 v/v), washed with H_2_O and NaCl-saturated solutions, and evaporated under reduced pressure. The resulting residue was redissolved in 4 mL of H_2_O/CH_3_OH (1:1 v/v) and purified using a PU 714 M pump, SC 762 system controller, and PLC 761 fraction collector (GL Sciences, Tokyo, Japan). The preparative column used was a TSKgel ODS-80Ts column (5 μm, 25 cm × 20.0 mm i.d., Tosoh Corporation). The elution conditions were as follows: mobile phase, H_2_O/CH_3_OH mixture containing 0.1% (v/v) FA; gradient elution (CH_3_OH % (min)): 20 (0–1), 70 (1–200), 95 (200–205), and 5 (205–210). The flow rate of the mobile phase was 5.0 mL/min. Purity (HPLC %) was determined to be >99% using a photodiode array detector (190–600 nm, ACQUITY PDA, Waters). The *m*/*z* of the peak was 275.0904 (theoretical value: 275.0910 [M + H^+^]), as determined by high-resolution MS (Xevo G2-XS QTOF, Waters). The analytical conditions and results of these purity tests are provided in the Supporting Information (Figure S1).

### Instrument Operating Conditions

Ultraperformance liquid chromatography–electrospray ionization–tandem mass spectrometry (UPLC–ESI–MS/MS) was performed using an ACQUITY UPLC H-class instrument (Waters, Milford, MA, USA) connected to a Xevo TQ-XS mass spectrometer (Waters, Milford, MA, USA). MS detection of the derivatives was performed in positive ESI mode using multiple reaction monitoring (MRM). The analytical columns used were an ACQUITY Premier BEH C18 column (1.7 μm, 100 × 2.1 mm i.d., Waters) or an ADME-HR column (2.0 μm, 100 × 2.1 mm i.d., Osaka Soda). A shorter ADME-HR column (2.0 μm, 50 × 2.1 mm i.d., Osaka Soda) was employed under optimal conditions. The elution conditions for the ACQUITY Premier BEH C18 column were as follows: mobile phase, a CH_3_CN/H_2_O mixture containing 0.1% (v/v) FA; gradient elution (CH_3_CN % (min)): 5 (0–0.5), 42 (0.5–16), 98 (16–18), and 5 (18–20). The flow rate of the mobile phase was 0.4 mL/min. The elution conditions for the ADME-HR column were as follows: mobile phase, a CH_3_CN/H_2_O mixture containing 10 mmol/L ammonium formate; gradient elution (CH_3_CN % (min)): 2 (0–0.3), 25 (0.3–7.5), 98 (7.5–8.5), and 2 (8.5–10). The flow rate of the mobile phase was 0.5 mL/min. The elution conditions for the shorter ADME-HR column were as follows: mobile phase, a CH_3_CN/H_2_O mixture containing 10 mmol/L ammonium formate; gradient elution (CH_3_CN % (min)): 2 (0–0.2), 39 (0.2–4.5), 98 (4.5–6), and 2 (6–7). Another set of conditions was also evaluated as follows: mobile phase, a CH_3_CN/H_2_O mixture containing 0.1% (v/v) FA; gradient elution (CH_3_CN % (min)): 2 (0–0.2), 25 (0.2–9), 98 (9–10), and 2 (10–11). The flow rate of the mobile phase was 0.5 mL/min. The MS/MS conditions are described in detail in the Supporting Information (Table S2).

**Figure 1:**
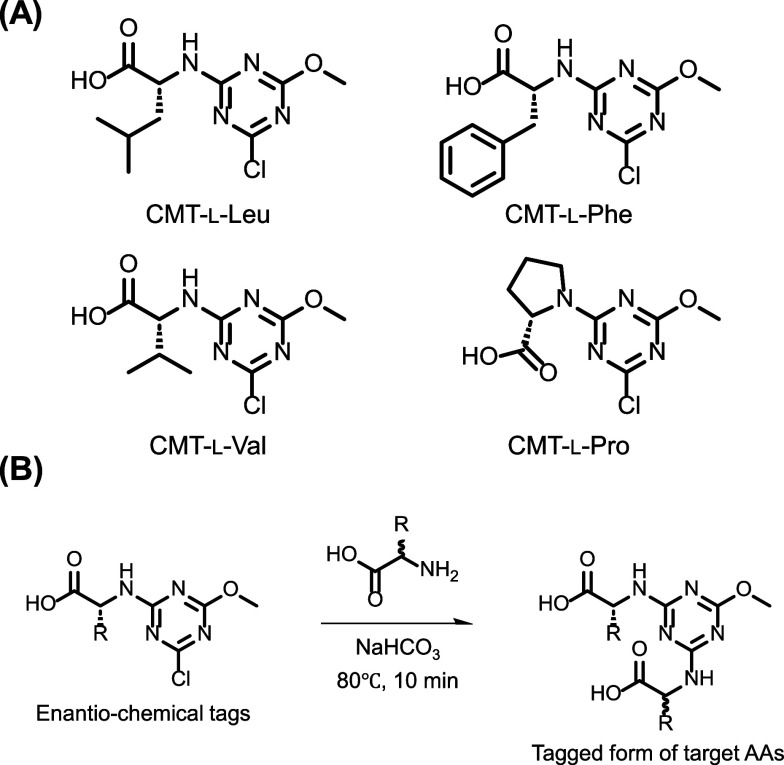
Structures of screened reagent candidates and their reaction. (A) Candidate structures and (B) reaction scheme.

**Table 1:** Chromatographic Resolutions of Target dl-AAs^a^

	structure of enantiochemical tagging site			
dl-AA	l-Leu	l-Phe	laQ-Pro	L-Val
Ala	5.98	6.99	0.83	4.90
Ser	2.45	2.72	1.05	2.27
Pro	1.44	3.12	1.39	1.86
Val	6.87	0.00	2.73	5.38
Thr	6.91	7.89	2.49	5.27
Ile	6.36	0.00	2.61	4.59
Leu	7.62	0.00	0.35	5.01
Asn	0.64	0.17	1.77	0.19
Asp	1.54	0.78	0.33	1.81
Gln	1.10	1.94	2.99	1.27
Lys	0.66	0.44	2.26	0.00
Glu	2.44	4.07	0.91	2.99
Met	6.17	6.21	1.99	4.88
His	0.64	0.29	0.45	0.54
Phe	8.26	3.12	2.08	3.83
Arg	1.69	1.69	2.40	1.60
Tyr	3.79	4.99	1.40	5.63
Trp	4.54	5.68	0.16	3.46

a Resolutions were calculated using the following equation: *R*_s_ = 1.18 × (*t*_RD_ – *t*_RL_)/(*W*_0.5D_ + *W*_0.5L_), where *t*_RD_ and *t*_RL_ are the retention times of the d- and l-form peaks, and *W*_0.5D_ and *W*_0.5L_ denote the half widths of the d- and l-form peaks.

**Figure 2:**
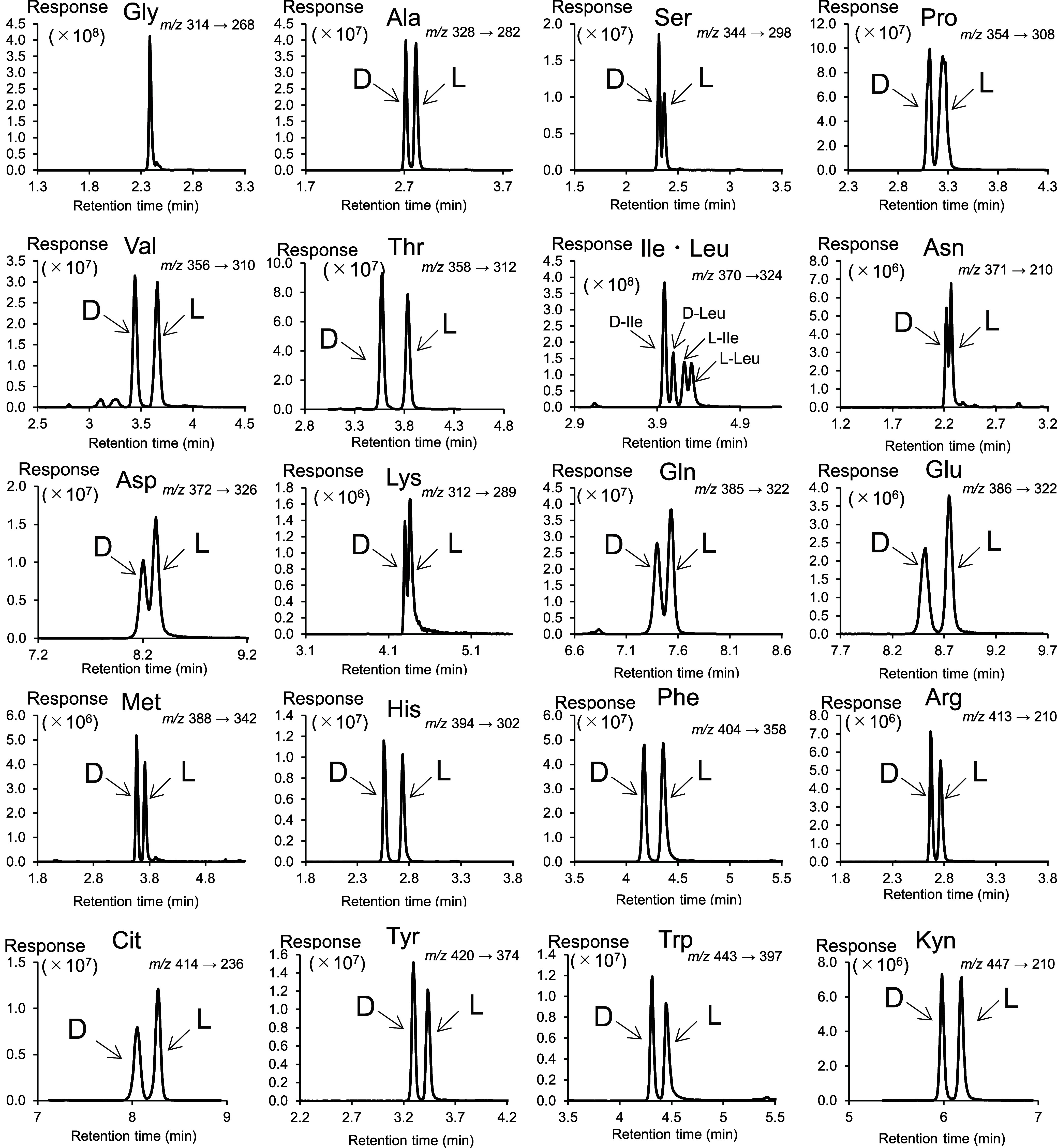
MS chromatograms under optimized conditions for 21 AAs, including enantiomers. The concentration of each enantiomer was set to 100 nmol/L in the vial.

**Figure 3:**
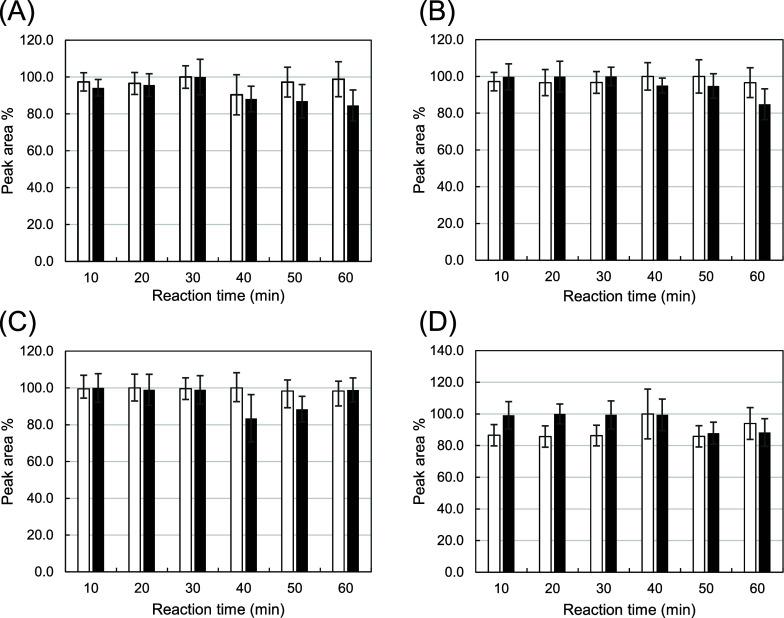
Comparison of representative reaction times for derivatization. Four representative AAs are shown: (A) Val, (B) Trp, (C) Lys, and (D) Glu. The outlined and solid black bars correspond to the results for l- and d-forms, respectively. The error bars indicate standard error (*N* = 3).

**Table 2:** Quantification Results of DSW Samples from Toyama Bay^a^

sample Lot		240415	250109	250519	Average	SE
Gly	Null	153	372	115	213	80
Ala	D	14.3	36.3	6.85	19.1	8.8
	L	213	491	155	286	104
Ser	D	10.1	30.3	19.6	20.0	5.8
	L	464	630	329	474	87
Thr	D	3.4	74.8	7.0	28.4	23.2
	L	128	150	98.0	125	15
Pro	D	2.16	7.85	7.95	5.99	1.91
	L	104	104	48.4	85.3	18.5
Val	D	11.0	27.0	4.51	14.2	6.7
	L	69.0	129	46.5	81.5	24.6
Leu	D	935	162	51.0	383	278
	L	439	192	404	345	77.2
Ile	D	3.58	7.65	NC	3.83	2.13
	L	22.9	54.5	13.5	30.3	12.4
Met	D	ND	2.07	ND	2.07	NA
	L	6.25	29.4	30.7	22.1	7.93
Glu	D	8.65	12.1	2.82	7.86	2.71
	L	91.5	108	114	105	7
Gln	D	6.90	9.85	NC	5.77	2.74
	L	27.2	28.9	28.4	28.1	0.5
Asp	D	5.00	1.44	2.95	3.13	1.03
	L	690	2775	945	1470	657
Asn	D	8.70	15.0	1.71	8.45	3.83
	L	58.0	40.5	30.4	42.9	8.1
Lys	D	20.6	24.9	ND	22.7	1.7
	L	121	69.5	ND	95.0	20.8
Arg	D	3.19	ND	3.26	3.21	0.04
	L	179	182	208	189	9
His	D	2.55	3.69	8.05	4.76	1.68
	L	79.5	108	85.5	90.8	8.5
Cit	D	ND	ND	ND	NA	NA
	L	121	120	72.4	105	16
Tyr	D	6.4	4.57	NC	3.91	1.66
	L	39.3	103	43.0	61.8	20.7
Phe	D	1.42	3.34	ND	2.38	0.78
	L	39.9	62.5	23.4	41.9	11.3
Trp	D	1.55	7.8	4.19	4.51	1.81
	L	ND	16.4	1.62	9.01	6.04
Kyn	D	ND	ND	ND	NA	NA
	L	NC	NC	NC	NA	NA

a The concentrations are expressed in nmol/L of DSW. *: Reference value (calculated using the calibration curve equation because the concentration exceeded the upper LOQ); NC: Not calculated because the value was below the lower LOQ; ND: Not detected; NA: Not applicable.

**Figure 4:**
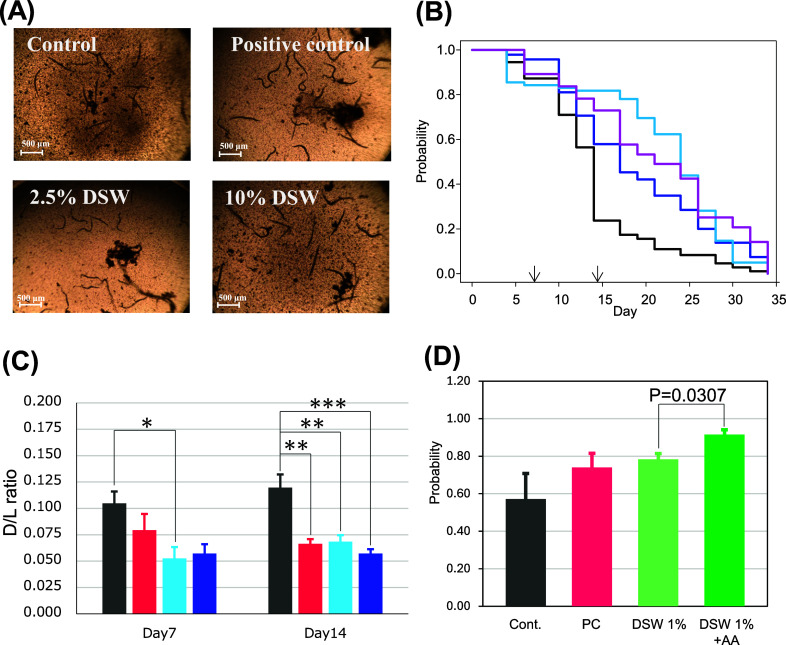
Lifespan assay of DSW using the *C. elegans* model. (A) Microscopic images of *C. elegans* observed on day 7. (B) Kaplan–Meier curve for each condition: black, control (Milli-Q water); pink, positive control (metformin); sky blue, 2.5% DSW-containing medium; and blue, 10% DSW-containing medium. The arrows indicate the time points at which *C. elegans* bodies were collected for dl-AA analysis. (C) dl-Asp analysis extracted from *C. elegans* bodies. The statistical analysis was conducted using Dunnett’s *t*-test, comparing each group with the control (*: *p* < 0.05, **: *p* < 0.01, ***: *p* < 0.005). (D) Simple lifespan assay to evaluate dl-AA contribution: black, control (Milli-Q water); pink, positive control (metformin); light green, 1% DSW-containing medium; and green, 1% DSW-containing medium diluted with dl-AA solution adjusted to the concentrations found in DSW. The statistical analysis was conducted using Tukey’s test for all combinations, and significant *p*-values are indicated in the graph.

### Derivatization Conditions of AAs

An aliquot of 10 μL of a 1 μmol/L dl-AA mixture was added to 10 μL of 20 mmol/L 2,4-dichloro-6-methoxy-1,3,5-triazine-d-Leu (CMT-d-Leu) in H_2_O/CH_3_CN = 1/1, followed by 10 μL of 100 mmol/L NaHCO_3_ in H_2_O. These mixtures were reacted for 10 min at 80 °C. After the reaction was completed, the mixture was evaporated under reduced pressure and redissolved in 100 μL of 1% FA in CH_3_CN/H_2_O (2:98, v/v).

### Validation of Quantification Analysis of dl-AAs in DSW

A mixture of 1900 μL DSW and 100 μL of dl-AA solutions at various concentrations (0–50 μM) was desalinated by adding it to 9 mL of CH_3_OH. These mixtures were centrifuged at 4000*g* for 5 min at 4 °C. The supernatant (1 mL) was then reacted with the reagent following the procedure described in the previous section. The resulting redissolved solutions were subjected to UPLC–ESI–MS/MS analysis. Quantitative analysis was conducted using the standard addition method to minimize interference from minerals. The calibration curve was constructed by plotting the peak areas (*y*) against the corresponding adjusted standard concentrations (*x*, nmol/L in the DSW sample). Regression analysis was performed using a weighted least-squares method with a weighting factor of 1/*x*. These steps were applied to each sample, and the resulting *y*-intercepts were divided by the corresponding slopes. The absolute values were reported as the concentrations of dl-AAs in the samples. Analytical method validation (linearity, limit of detection (LOD), limit of quantification (LOQ), and intra- and interday reproducibility) was conducted in accordance with the ICH-M10 guideline (Bioanalytical Method Validation and Study Sample Analysis). A recovery test from DSW was performed at three concentrations. Intra- and interday precisions were assessed by quantifying dl-AAs in DSW over 3 d (N = 3 per day). Furthermore, the matrix effect and reaction recovery at a single concentration were evaluated to assess potential interference from DSW. Details of the validation protocol are provided in the Supporting Information.

### Lifespan Assay Using *C. elegans* Model

The lifespan assay was conducted according to the method described in a previous study.^25^ Briefly, the wild-type nematode *C. elegans* (Bristol strain N2) was used as a model organism. *C. elegans* stocks were maintained on nematode growth medium (NGM) plates seeded with 50 μL *E. coli* (DH5αFT) at 20 °C as a food source. Eggs were collected from *C. elegans* by lysing them with KOH and NaClO. The eggs were then left in Milli-Q water overnight to synchronize *C. elegans* at the L1 stage. After the worms reached L1, they were transferred onto NGM plates with *E. coli* until the L4 stage. On the third day, 2 mmol/L of 5-fluoro-2′-deoxyuridine (FUDR; FUJIFILM, Miyazaki, Japan) was added to suppress reproduction. On the fourth day, *C. elegans* were exposed to liquid culture medium composed of 75 μL of FUDR, 550 μL of S-basal, 1.5 μL of 2% cholesterol, 75 μL of solvent (DSW, 25% DSW, 500 mM metformin, or Milli-Q water as a control), and 50 μL of Milli-Q water containing *C. elegans*. The lot number of DSW used in this study was 250109. Next, 750 μL of this liquid culture was added to each well of a 24-well plate containing a cell culture insert with a 3 μm translucent polyethylene terephthalate (PET) membrane (ThinCerts; Greiner Bio-One, Kremsmünster, Austria). The membrane functioned as a net to retain the worms while allowing diffusion of liquid culture components. Each well contained approximately 30 worms, with *N* = 3 wells per group for each assay. In addition, 500 μL of liquid culture outside the inset was replaced three times per week. The number of live worms was also recorded three times per week. The worms were considered dead if they showed no movement. Samples of *C. elegans* were collected on days 0, 7, and 14 after exposure. *C. elegans* were collected from the wells and diluted to 400 μL. The samples were crushed with beads (3 g of 3 mm beads and 0.7 g of 1 mm beads) using a Multibeads Shocker (YASUI KIKAI, Osaka, Japan). Subsequently, 200 μL of supernatant was added to 800 μL of CH_3_CN for deproteinization by centrifugation at 10,000*g* for 5 min at 4 °C. Next, 800 μL of the resulting supernatant was evaporated under reduced pressure and redissolved in 100 μL of CH_3_CN/H_2_O (1:1, v/v). Finally, 50 μL of the redissolved solution was reacted with CMT-d-Leu and analyzed following the procedures described in the previous sections. These processes were replicated twice, and the results were analyzed by combining the data from both replicates. The total number of individuals for each well is shown in Figure S2.

### Contribution of dl-AAs in DSW to the Lifespan Extension of *C. elegans*

To examine the contribution of dl-AAs in DSW, the lifespan extension experiment was conducted following the method described in the previous section, with two modifications: the solvent used in the liquid culture medium and the number of worms per well. For each well, 75 μL of solvent was added, which consisted of either 10% DSW, 10% DSW supplemented with the dl-AA mixture, 500 mM metformin, or Milli-Q water (control). The concentration of the dl-AA mixture was prepared based on the quantification results of DSW. Each well contained approximately 20 worms, and each group was tested in six independent assays.

## Results and Discussion

### Screening of Reagent Structure for dl-AA Separation

Chiral derivatization reagents were synthesized and evaluated to develop a rapid method for screening dl-AAs. DMT-(*S*)-Pro-OSu is a reagent that enables selective detection of dl-AAs with high sensitivity, owing to enhanced ESI efficiency by the triazine moiety.^26^ However, this method requires five different LC modes and a total of 40 min to complete detection. In this study, a novel reagent was designed and developed. Bhushan and Kumar previously developed several chlorotriazine-type chiral derivatization reagents for dl-AA separation,^27^ achieving moderate separation speed in the standard LC mode. Therefore, we aimed to enhance separation efficiency by modifying the optically active sites of the reagent. Figure 1 shows the screened structures of the candidate reagents. All the reagents possess an asymmetric carbon at the α-AA moiety. Semipurified reagents obtained using pTLC were employed to evaluate the separation efficacy for all the proteogenic dl-AAs. Table 1 compares the chromatographic resolutions of enantiomers for all the target compounds under preliminary conditions. The best separation was achieved using derivatives of the CMT-l-Leu reagent. Under these conditions, the target l-AAs eluted earlier than the d-AAs, which is undesirable because the concentrations of d-AAs in the samples are expected to be lower than those of l-AAs. In principle, the retention order can be reversed by inverting the asymmetric carbon in the reagent moiety; therefore, CMT-d-Leu was selected as the optimal reagent.

Figure S3A shows the MRM chromatograms of dl-AA separation using CMT-d-Leu with conventional octadecylsilane-type columns under FA acidic conditions. All the targeted dl-AAs were separated, except for Asn and His. Increasing the basicity of the mobile phase partially resolved this issue; however, Asp and Glu remained unresolved (Figure S3B). Therefore, further separation was performed by varying the column type. In a previous study on DMT-(*S*)-Pro-OSu, an ADME column was employed, which showed an improved retention pattern compared with a conventional column. Similarly, an ADME column was used in this study for dl-AA separation. Figure S4A shows the MRM chromatograms obtained under acidic conditions, indicating insufficient separation of Asn and His. In contrast, as shown in Figure S4B, good separation was achieved for all other targets under 10 mmol/L ammonium formate conditions. Although all the targeted dl-AAs were fully separated within 10 min per run, the peak shapes of Asp, Glu, and Lys exhibited tailing. Their derivatives contain tricarboxylic acid groups, indicating that stronger interactions under basic conditions impaired peak performance compared with a 0.1% FA-containing mobile phase. This issue likely compromised the quantification and/or robustness of column performance. Therefore, a shorter ADME column (50 mm) was employed to improve throughput, and a separation method using two divided elution conditions was developed within a single analytical system (Figure 2). Under this condition, the nonproteogenic dl-AAs, Cit and Kyn, were well separated. The LOQ, defined as the concentration with *S*/*N* = 10, was determined. LOQ values of 0.33–54.9 pmol/L on column (in vial) were obtained under the final conditions (Table S3). These sensitivities were sufficient to detect rare AAs in DSW. Based on these results, a high-throughput, highly sensitive method using the optimal reagent, CMT-d-Leu, was developed.

### Optimization of the Reaction Conditions and Validation

The reaction conditions for dl-AAs were optimized in water with a high mineral content for DSW analysis. Initially, interference from the minerals in DSW hindered the reaction (data not shown). Therefore, a method was developed to reduce the mineral content in DSW. We considered the differences in solubility between the minerals and AAs. When nine times the sample volume of methanol was added, significant precipitation was observed in the sample tube. Figure 3 shows the reaction time course from 10 to 60 min for five representative AAs. A 100 mmol/L NaHCO_3_ solution (pH 12) was used as the basic catalyst. The maximum and plateau responses were reached after 10 min. The reagent was highly water-soluble, even with tagged dl-AAs, suggesting that reactions in a water-rich matrix could be achieved using this novel technology. The recovery rate of AAs from the DSW sample was examined under these reaction conditions. Table S4 shows the recovery rates and precision. Spiking concentrations were set at 25, 250, and 1500 nmol/L for the l-AAs, including Gly, and 5, 50, and 300 nmol/L for the d-AAs. The results indicate that the spiked concentrations were fully recovered using the standard spiking calibration method. The recovery and matrix effect in DSW were also assessed. As shown in Table S5, approximately 60–70% of AAs were recovered from the spiked DSW samples, and no significant matrix effects were observed for any AAs (0.85–1.15). The negligible matrix effect can likely be attributed to the substantial difference in retention time between minerals, presumed to elute near t_0_, and the chemically tagged forms of dl-AAs. These validation results demonstrate that accurate quantification of dl-AAs in DSW can be achieved using the proposed method.

### Quantification Results of DSW

Based on the results described in the previous section, a quantitative analysis of DSW was performed using the established method. Table 2 shows the quantification results for DSW samples from Toyama Bay. The samples were analyzed three times over the course of a year to determine the stability of the DSW content. Contrary to the initial expectation that dl-AA concentrations would remain stable throughout the year, notable variations were observed among the three samples. This variability can likely be attributed to differences in mountain water inflow from the Southern Alps of Japan. Further investigation of Toyama Bay DSW, along with comparative analyses at additional sampling sites, is needed to verify this hypothesis. Relatively high concentrations of d-Leu, d-Val, d-Ala, d-Thr, and d-Ser were quantified. Lower concentrations of d-Glu, d-Asn, and d-Pro were also detected in DSW. These results suggest that the AA content profile varies with ocean water depth. To the best of our knowledge, this is the first study to quantify dl-AAs in DSW from Japan. Different effects of d-AAs have been reported; therefore, these results provide an interesting perspective for explaining the efficacy of DSW and/or its concentration. The higher concentration of d-AAs in DSW has been suggested to result from marine snow.^28^ Marine snow forms during the death cycle of plankton, marine bacteria, and other organisms such as fish. Most marine snow is thought to contain the remains of bacteria and plankton, suggesting that its composition includes various types of prokaryotes.^29^ Prokaryotes generally use d-AAs for initiation and production. One study suggested that d-AAs are selectively used by deep-sea microorganisms.^14^ In addition, d-Asp, d-Glu, and d-Ala have been detected in seabed hydrothermal sediments from the Izena and Yoron Cauldrons, Okinawa Trough.^30^ L-Kyn in DSW was detectable (>LOD) but remained below the LOQ, which is consistent with the level from the previous report.^10^ Although Kyn may have important biological effects, it is unlikely to exert the same effects in our nematode assay owing to species-specific differences between fish and *C. elegans*. These findings suggest that dl-AA profiles may be suitable for analyzing marine areas, and their measurement is valuable not only for mechanistic studies of DSW but also for assessing the specificity of the ecosystem.

### Lifespan Assay of *C. elegans* with DSW Addition and Analysis of dl-AAs in the Body

Finally, the mechanistic contribution of DSW was investigated using the *C. elegans* model. Several studies have indicated that the beneficial effects of DSW require continuous intake.^31^ *C. elegans* was used in this study as an animal model to evaluate lifespan during screening. The nematode *C. elegans* is a small organism with a shorter lifespan than higher-order animals such as mice. In addition, it has few organs and a genome similar to that of humans. Therefore, this small organism is considered suitable for use in lifespan assays employing a simple experimental system, such as a well chamber. Previous studies using the Transwell port system to facilitate daytime treatments during the experimental schedule were referenced.^32,33^ Figure 4A shows microscopic images of *C. elegans*, and Figure 4B shows the resulting lifespan analysis curve. The movements of living organisms were clearly observed under the microscope. Observations throughout the lifespan revealed a significant prolongation of lifespan in the positive control and DSW-treated samples. Table S6 shows the results of log-rank tests applied to the Kaplan–Meier survival curves. Pairwise comparisons indicate significant differences between the control and each of the other groups (*p* < 0.05), whereas no significant differences were observed among the treatment groups. These results suggest that DSW demonstrates antiaging efficacy in the *C. elegans* model, equal to or greater than the positive control. Given that the AA concentrations were lower than those of the mineral components, their contribution to the observed lifespan extension may be limited. The contributions of dl-AAs to lifespan prolongation were not directly evident in this study, particularly considering the expected amounts of proliferative AAs in DSW.^25^

dl-AAs in *C. elegans* bodies were further examined using the developed dl-AA analytical system to evaluate their absorption from DSW and their potential as aging biomarkers. Figure 4C shows the analytical results for dl-Asp, which was the only AA exhibiting significant changes on day 14. The d/l ratio significantly decreased in the treated groups on day 14, with a similar decreasing trend observed on day 7. d-Asp has attracted considerable attention as an aging marker derived from the aging reaction of the proteogenic l-Asp moiety.^34,35^ These results suggest that d-Asp may serve as a rapid biomarker for aging in *C. elegans*. In addition, fluctuations in d-Asp could potentially provide a faster marker for lifespan assays. Further validation studies may establish d-Asp as a rapid biomarker for such assays. The accumulation of AAs in the bodies was not observed in this analysis. Therefore, to elucidate the contribution of dl-AAs in DSW to the antiaging effect, further research comparing the effects of adding purified dl-AAs to DSW with those of water-diluted DSW was conducted. Because the results indicated that the median survival time in the control was approximately day 14, this additional investigation focused on the viability at day 14. To highlight the effect of dl-AAs, a 1% DSW group, which corresponds to the concentration at which lower effects were observed, was prepared alongside the dl-AA addition group. As shown in Figure 4D, the dl-AA addition sample exhibited a significant recovery effect on prolongation. This result suggests that the dl-AA profile in DSW may partially contribute to cell prolongation, either independently of or in complement to the effects of minerals. Insulin/IGF-1 signaling and oxidative stress resistance have been reported^36,37^ as components of the prolongation mechanism of DSW. Both signals are well established in *C. elegans* and other animals as factors influencing longevity.^38^ We plan to conduct mechanistic studies to investigate molecular biological processes, including in-house metabolomics and assays of signaling factor responses.^39,40^

## Conclusions

In this study, a rapid dl-AA screening method was developed using a novel enantiochemical tagging reagent, CMT-d-Leu. Screening of reagents and columns enabled a 14 min gradient elution with effective separation of 19 AAs for quantitative analysis. Rapid tagging of target AAs was completed within 10 min of reaction, suggesting that this novel technology is suitable for rapid and easy analysis. The detection sensitivity under optimized conditions ranged from 10 to 100 pmol/L (LOD). The reagent demonstrated superior performance compared with previously reported reagents for enantioselective analysis (Table S7). Furthermore, CMT-d-Leu and its tagged forms exhibit good water solubility, making them preferable for reactions in aqueous samples, such as seawater. Moreover, the sensitivity is sufficient to detect dl-AAs in seawater, enabling quantification of several dl-AAs at 10–100 nmol/L. The content profiles of dl-AAs in DSW can be used for mechanistic analysis and environmental research in marine studies. Although improvements to the method are still needed, such as expanding targets to include allothreonine and highly reactive thiol AAs such as cysteine, this novel tagging reagent shows promise for the rapid separation of additional targets. In addition, biological analysis using *C. elegans* revealed the proliferative effects of DSW. Although concentrated d-AAs in DSW were not detected in the *C. elegans* body, the aging marker d-Asp significantly decreased in the proliferated groups. This suggests that DSW reduced aging in *C. elegans*, similar to the positive control. The contribution of dl-AAs in DSW was further investigated by comparing the effects of adding purified dl-AAs to DSW with those of water-diluted DSW. The assay revealed that the dl-AAs contributed to the proliferative effect of DSW. However, this evaluation only examined the indirect relationship between dl-AAs and this effect. An in-depth mechanistic study is being planned to investigate the effects of DSW on proliferative signals such as insulin/IGF-1 signaling and oxidative stress resistance.

## Supplementary Material



## Data Availability

The data sets generated and/or analyzed during this study are available from the corresponding author upon reasonable request.

## Notes

Additional methods and data, including detailed LC–MS/MS conditions, well plate information, purity test results, MRM chromatograms, LLOQ and validation results, significance tests of the lifespan assay, and comparison of chiral derivatization reagnet's peformance (PDF)
