# Supplementary material for: Accelerated dl-Amino Acid Quantification of Deep Sea Water from Toyama Bay and Anti-Aging Activity Investigation Using Caenorhabditis elegans
Source: Anal Chem. 2026 Mar 11;98(11):8394–403. doi: 10.1021/acs.analchem.5c07605 (PMC13019432; doi:10.1021/acs.analchem.5c07605)
Supplement: Supplementary file 1 [file ac5c07605_si_001.pdf]

Supporting Information

Accelerated DL-Amino Acid Quantification of Deep Sea Water from Toyama Bay and Anti-Aging Activity Investigation Using *Caenorhabditis elegans*

Takahiro Takayama[1†]*, Haruto Iwata[1†], Reina Fujio[1], Ayaka Minamida[1], Yuko Sakaguchi[2], and Koichi Inoue[1]

1 Laboratory of Clinical & Analytical Chemistry, College of Pharmaceutical Sciences, Ritsumeikan University, 1-1-1 Nojihi-gashi, Kusatsu, Shiga 525-8577, Japan
2 College of Pharmaceutical Sciences, Ritsumeikan University, 1-1-1 Nojihi-gashi, Kusatsu, Shiga 525-8577, Japan

** Author for correspondence: Takahiro Takayama, Assistant Prof., Ph. D.*
Laboratory of Clinical & Analytical Chemistry, College of Pharmaceutical Sciences, Ritsumeikan University, 1-1-1 Nojihigashi, Kusatsu, Shiga 525-8577, Japan
E-mail address: t-takaym@fc.ritsumei.ac.jp (T. Takayama)

Table of Contents

Purity test for 2,4-Dichloro-6-methoxy-1,3,5-triazine-D-Leu 3
Additional instrumental conditions. 4
Method validation (Matrix effect and Reaction recovery from DSW). 5
Figure S1. Purity test results of (A) HPLC assay (190-600 nm), (B) mass spectrum of the peak eluted at 25.3 min, and (C) HPLC assay (254 nm) using the chiral column. 6
Figure S2. Twenty-four well information on life span assay using *C.elegans*. 7
Figure S3. MS chromatograms of D-Leu-modified reagents using BEH-C18 column with (A) 0.1% formic acid mobile phase and (B) 10 mmol/L ammonium formate. 8
Figure S4. MS chromatograms of D-Leu-modified reagents using CAPCELL PAK ADME-HR with (A) 0.1% formic acid mobile phase and (B) 10 mmol/L ammonium formate. 9
Figure S5. MS chromatograms of analytical results in DSW lot No. 240415. 10
Figure S6. MS chromatograms of analytical results in DSW lot No. 250109. 11
Figure S7. MS chromatograms of analytical results in DSW lot No. 250519. 12
Table S1. Optical purity of the DL-AAs used in this study. 13
Table S2. The optimized detection condition of MS/MS. 14
Table S3. The LOQ values in optimization conditions. 15
Table S4 Analytical method validation results of DSW as of recovery rate and repeatability 16
Table S4 Analytical method validation results of DSW as of recovery rate and repeatability (Continues)... 17
Table S4 Analytical method validation results of DSW as of recovery rate and repeatability (Continues)... 18
Table S5. Recovery rate evaluation with chemical tagging reaction and matrix effect from DSW. 19
Table S6. The result of log-rank test of *C.elegans* life span assay. 21
Table S7. The comparison of the performance of the entantio-chemical tags. 22

Purity test for 2,4-Dichloro-6-methoxy-1,3,5-triazine-D-Leu

The purity test of tag was performed using an ACQUITY UPLC H-Class PLUS system (including PDA) coupled to a quadrupole time-of-flight mass spectrometer (QToF-MS, Xevo G2XS QTOF, Waters, Milford, MA). The LC conditions were as follows: Column temperature: 40 °C, flow rate: 0.4 mL/min, Injection volume: 1 µL, mobile phase A: 0.1% FA in water, mobile phase B: 0.1% FA in acetonitrile, gradient programs: phase A/B (%): 95/5 (0–0.5 min) → 5/95 (55.0–57.0 min) → 95/5 (57.1–60.0 min), PDA range: 190 nm–600 nm. The MS system was equipped with an ESI source operating in positive ion mode. Nitrogen was used for the nebulization and desolvation (N_2 Supplier Model T30F, Anest Iwata, Yokohama, Japan), and argon was used as the collision gas. The column temperature was 40°C. The ESI source conditions included a capillary voltage of 2.50 kV, source temperature of 150°C, and desolvation temperature of 400°C. The cone and desolvation gas flows were 50 L/hr and 800 L/hr, respectively. The collision gas flow rate and was regulated at 0.15 mL/min. For optical purity measurement, CHIRALPAK IA-3 (3 µm, 2.1 mm × 150 mm; Daicel Corp., Osaka, Japan) was equipped for enantio-separation of CMT-D-Leu. The LC conditions were as follows: Column temperature: 40 °C, flow rate: 0.2 mL/min, Injection volume: 5 µL, mobile phase A: 0.1% FA in water, mobile phase B: 0.1% FA in acetonitrile, elution; 40% as isocratic B(%), detection; UV-vis 254 nm. The all solution for the analysis was prepared as a concentration of 2 mmol/L diluted by the initial mobile phases.

Additional instrumental conditions.

The MS system (Xevo TQ-XS) was equipped with an ESI source operating in positive ion mode. Nitrogen was used for the nebulization and desolvation (Oilfree Scroll SmartAir SLP-221EFD, Anest Iwata, Yokohama, Japan), and argon was used as the collision gas. The ESI source conditions included a capillary voltage of 2.50 kV, source temperature of 150°C, and desolvation temperature of 500°C. The cone and desolvation gas flows were 150 L/hr and 1000 L/hr, respectively. The collision gas flow rate was regulated at 0.19 mL/min. The LH resolution 1, HM resolution 1, ion energy 1, LM resolution 2, HM resolution 2, and ion energy 2 were 2.8, 15.1, 0.5, 2.7, 14.7 and 0.9, respectively. The dwell time was automatically set by the software.

The MRM transitions with detection energies were optimized before the analysis. These values were summarized in Table S1

Method validation (Matrix effect and Reaction recovery from DSW).

Matrix effect

A pair of evaporated residues from 10 µL of 5000 nmol/L DL-AAs tagged standards was prepared according to the chemical tagging method. One of them was redissolved as the usual method (Ref sample). Similarly, the DSW (mixture of 3 lots) derivatized samples were prepared, and the resulting solution was added to the other residues (Matrix sample). The only DSW derivatized sample was also prepared (Base sample), and these processes were replicated 6 times. All 18 samples were subjected to the LC-MS/MS analysis described in the “Instrumental conditions” section.

The matrix effect (ME) was calculated following the equation;

$ME = (Matrix_{peak\ area} - Base_{peak\ area})/Ref_{peak\ area}$

Where $Matrix_{peak\ area}$ indicates the peak area from the Matrix sample, $Base_{peak\ area}$ indicates the peak area from the Base sample (to eliminate interferences of DSW contained DL-AAs), and $Ref_{peak\ area}$ indicates the peak area from the Ref sample. The averages of the MEs and RSD% of N=6 analyses are summarized in Table S5.

Reaction recovery

The DL-AAs standards spiked sample to the DSW (mixture of 3 lots, spiked concentration at 500 nmol/L) was prepared (Spiked sample, N=6). Additionally, non-spike DSW (mixture of 3 lots) sample was prepared (Base sample, N=6). These samples were quantified by using a standard calibration curve (1-1000 nmol/L, 8 points) without spiking to DSW. All 20 samples were subjected to the LC-MS/MS analysis described in the “Instrumental conditions” section.

The reaction recovery rate (RR%) was calculated following equation;

$RR\% = (Spiked_{concentration} - non\text{-}Spiked_{concentration})/Theoretical_{concentration} \times 100$

Where $Spiked_{concentration}$ indicates the concentration from the quantification with a standard calibration curve, $non\text{-}Spiked_{concentration}$ indicates the concentration from the DSW quantification (to eliminate interferences of DSW contained DL-AAs), and $Theoretical_{concentration}$ indicates the concentration of the theoretical value (500 nmol/L). The averages of the RRs and RSD% of N=6 analyses are summarized in Table S5.

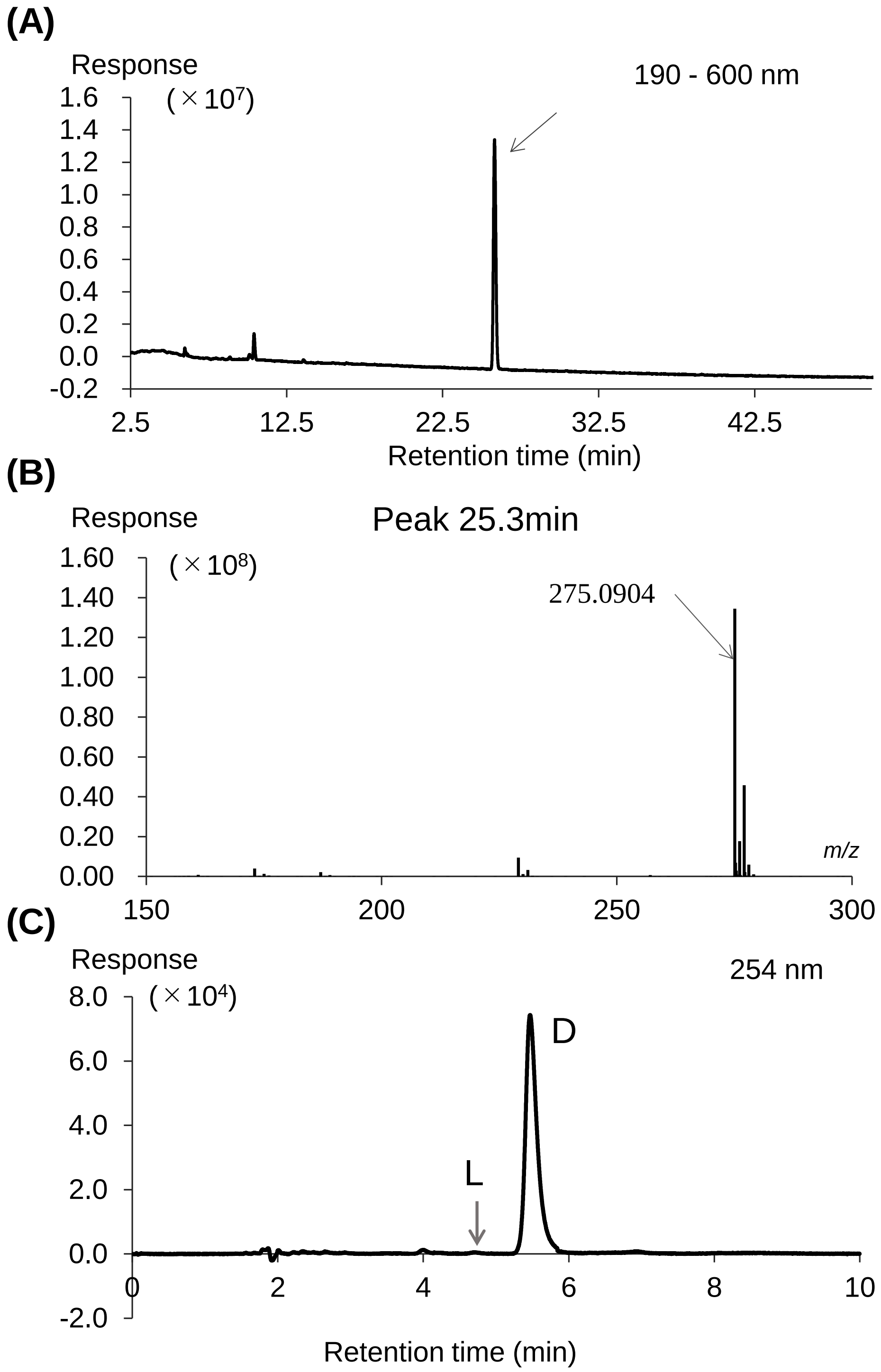

Figure S1. Purity test results of (A) HPLC assay (190-600 nm), (B) mass spectrum of the peak eluted at 25.3 min, and (C) HPLC assay (254 nm) using the chiral column.

The chemical purity of the tag was calculated as 98.2% (area, %). The optical purity of the tag was calculated as 99.2 ee% (area, %). The elution position of the L-isomer of the tag was identified by the analysis of the CMT-L-Leu tag.

Life span assay of DSW

Batch 1

	Cont.	PC	DSW 2.5%	DSW 10%		
1	35	43	34	36		
2	35	30	31	37		
3	44	28	32	40		

Batch 2

1	31	42	33	31		
2	38	27	27	31		
3	38	22	27	34		

Expansion study

	1	2	3	4	5	6
Cont.	23	12	16	19	22	22
PC	16	21	17	22	20	20
DSW1%	31	28	18	23	20	22
DSW1% +AA	22	21	26	23	25	31

Figure S2. Twenty-four well information on life span assay using *C.elegans*.

The number in the well indicates the individual number of C.elegans. PC: positive control (metformin).

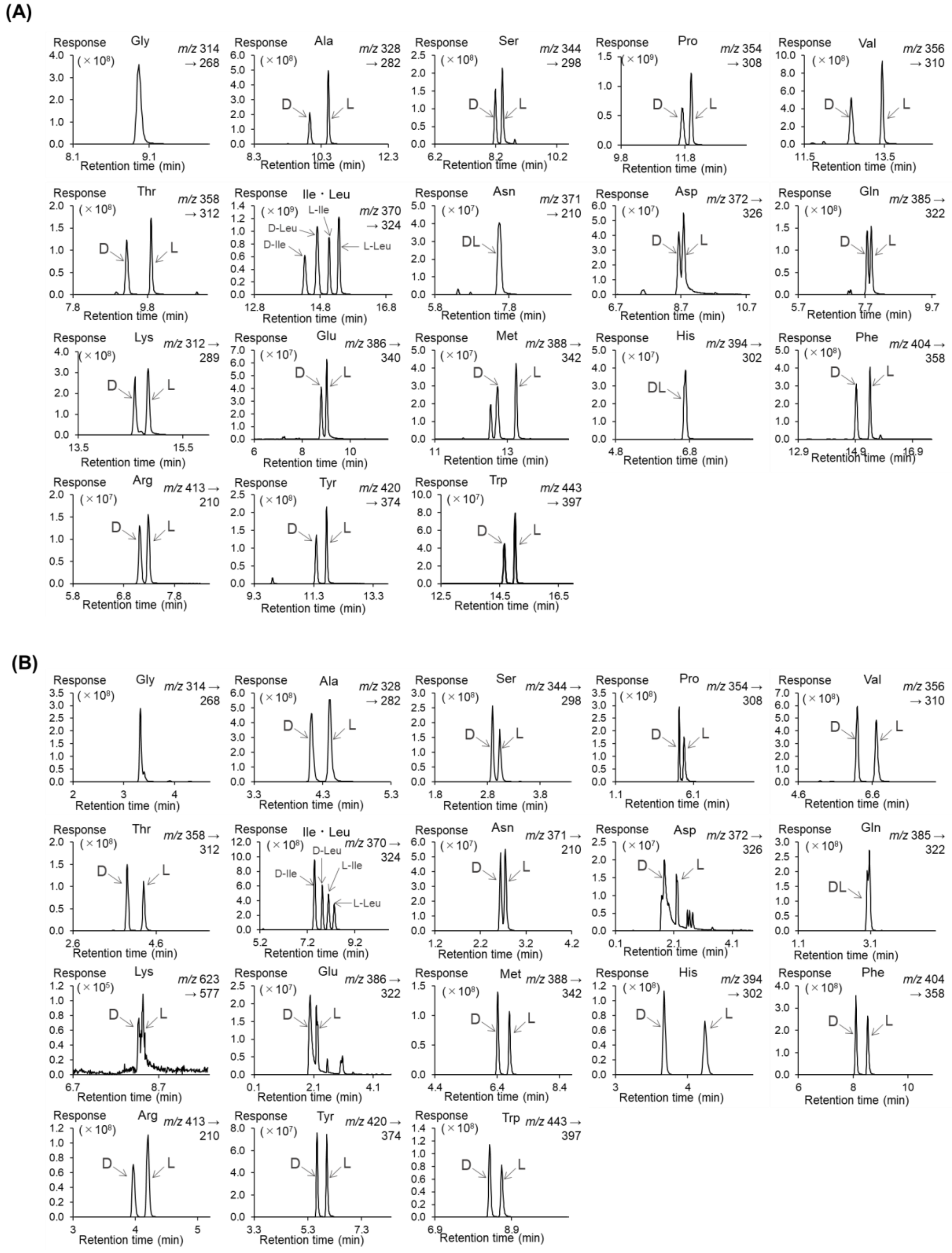

Figure S3. MS chromatograms of D-Leu-modified reagents using BEH-C18 column with (A) 0.1% formic acid mobile phase and (B) 10 mmol/L ammonium formate.

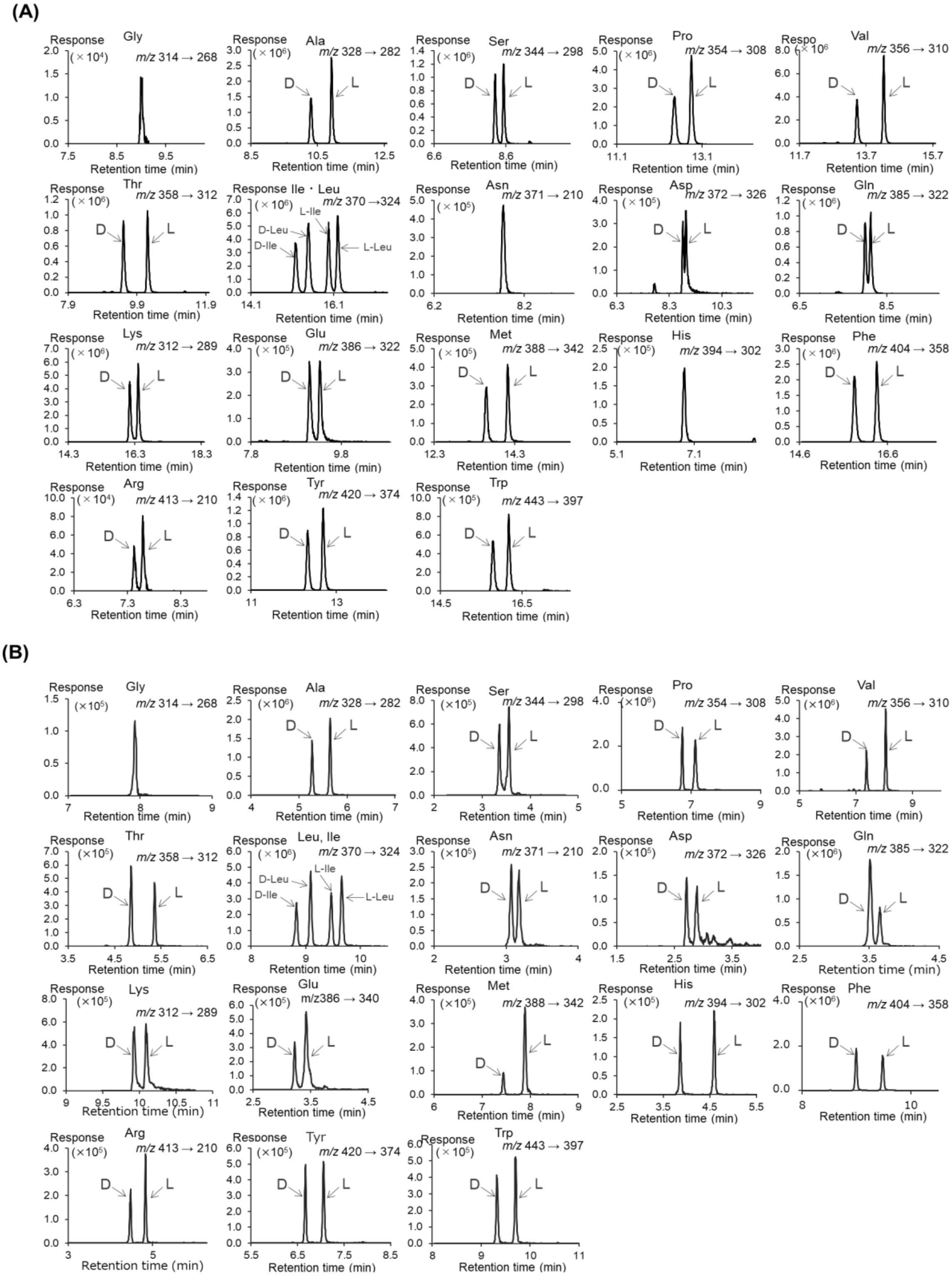

Figure S4. MS chromatograms of D-Leu-modified reagents using CAPCELL PAK ADME-HR with (A) 0.1% formic acid mobile phase and (B) 10 mmol/L ammonium formate.

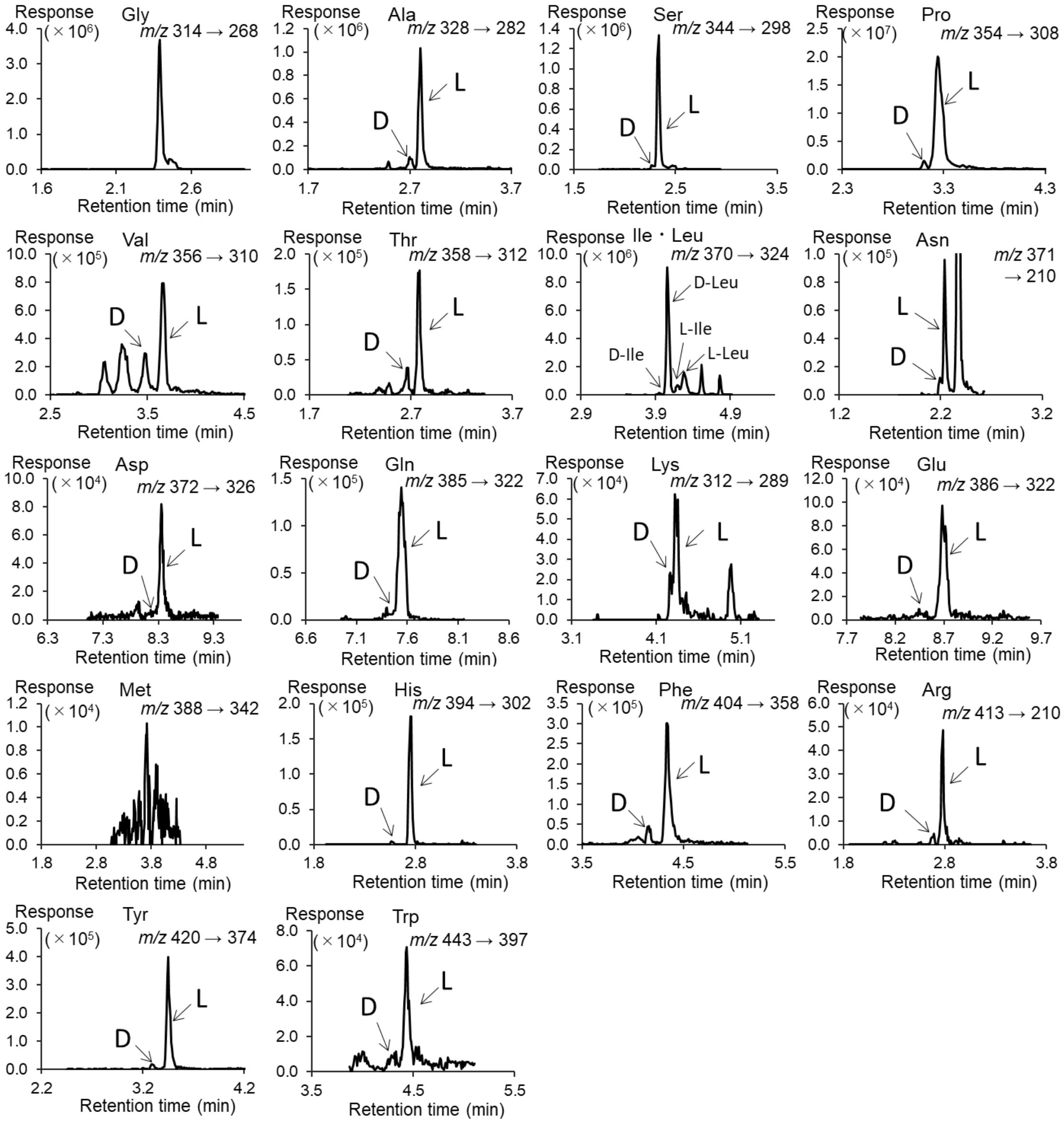

Figure S5. MS chromatograms of analytical results in DSW lot No. 240415.

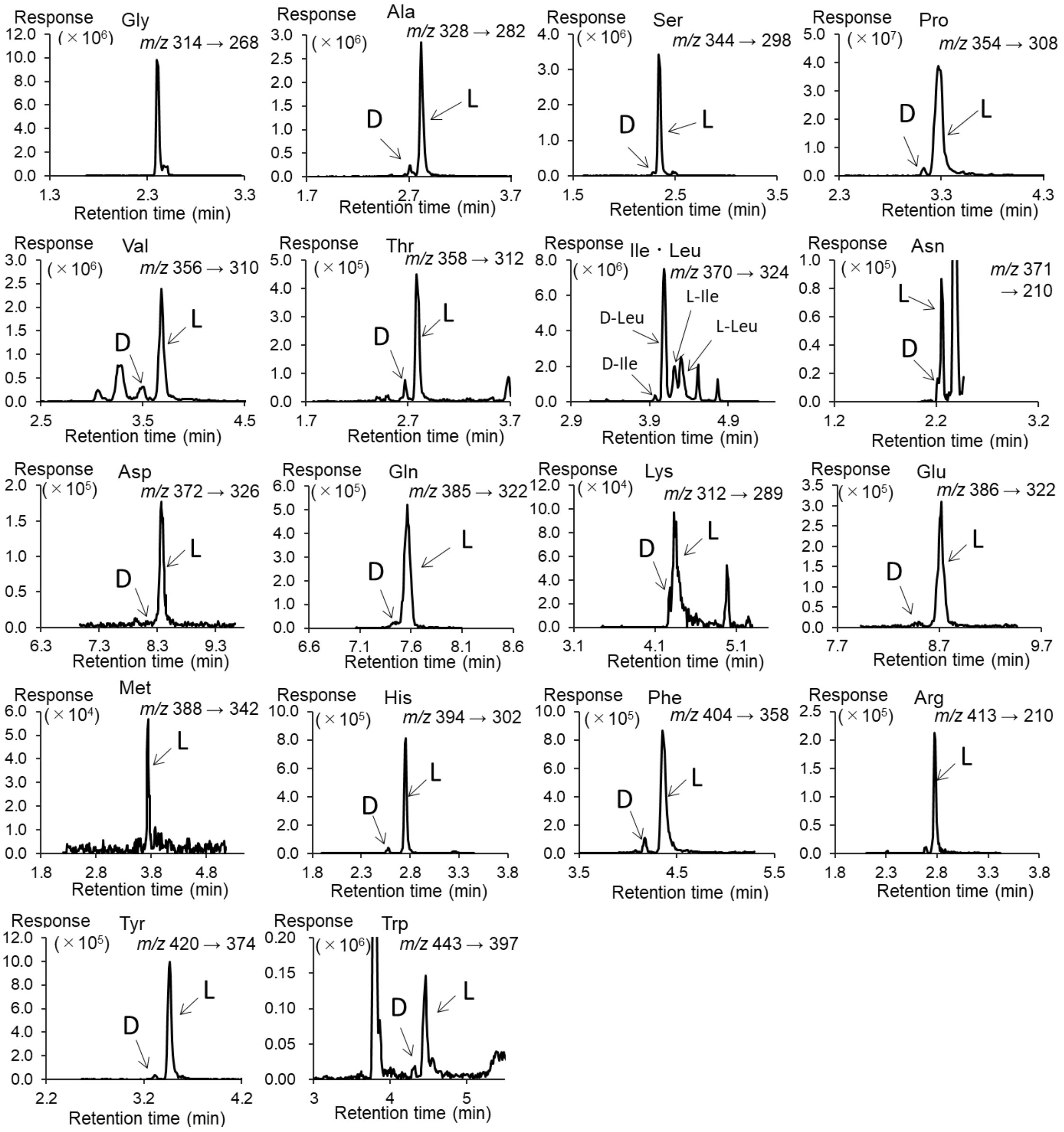

Figure S6. MS chromatograms of analytical results in DSW lot No. 250109.

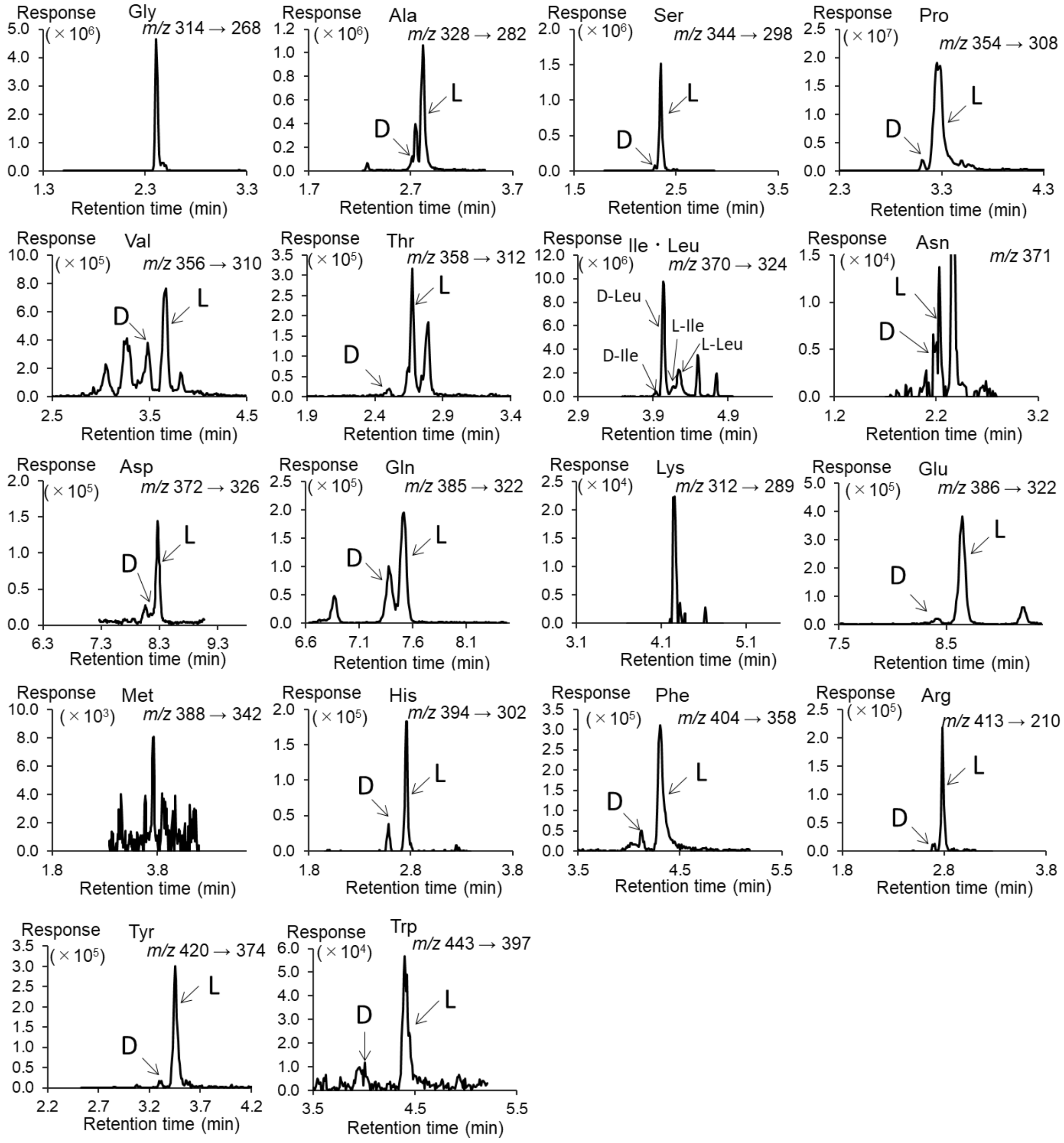

Figure S7. MS chromatograms of analytical results in DSW lot No. 250519.

Table S1. Optical purity of the DL-AAs used in this study.

Analytes (Abbreviation)	D-form (ee%)	L-form (ee%)
Ala	100.0	98.6
Ser	98.4	100.0
Pro	99.4	100.0
Val	100.0	100.0
Thr	100.0	100.0
Ile	100.0	100.0
Leu	99.5	100.0
Asn	99.8	100.0
Asp	100.0	100.0
Gln	100.0	100.0
Lys	100.0	100.0
Glu	100.0	100.0
Met	99.3	100.0
His	100.0	100.0
Phe	100.0	100.0
Arg	97.8	100.0
Cit	98.0	100.0
Tyr	100.0	100.0
Trp	98.4	100.0
Kyn	99.9	99.8

Table S2. The optimized detection condition of MS/MS.

Analytes (Abbreviation)	Monitoring ions (m/z)	Cone voltage (V) /Collision energy (eV)
Gly	314 > 268	40/30
Ala	328 > 282	40/20
Ser	344 > 298	40/20
Pro	354 > 308	40/20
Val	356 > 223	40/20
Thr	358 > 312	40/20
Ile	370 > 324	40/20
Leu	370 > 324	40/20
Asn	371 > 210	40/31
Asp	372 > 326	40/20
Gln	385 > 322	40/24
Lys	312 > 289	40/15
Glu	386 > 340	40/21
Met	388 > 342	40/20
His	394 > 302	40/30
Phe	404 > 358	40/20
Arg	413 > 210	40/40
Cit	414 > 236	40/40
Tyr	420 > 374	40/25
Trp	443 > 397	40/20
Kyn	447 > 210	40/35

Table S3. The LOQ values in optimization conditions.

Analyte	Calibration range (nmol/L)	LOQ (pmol/L)	R^2
Gly	1-2500	0.33	0.999
Ala	1-2500	4.3	0.999
Ser	1-2500	9.2	0.999
Thr	1-2500	2.8	0.997
Pro	1-2500	1.0	0.992
Val	1-2500	4.0	0.994
Leu	1-2500	2.7	0.996
Ile	1-2500	1.4	0.997
Met	1-2500	30.5	0.993
Glu	1-2500	20.8	0.998
Gln	1-2500	1.8	0.999
Asp	1-2500	11.1	0.996
Asn	1-2500	54.9	0.993
Lys	1-2500	54.9	0.998
Arg	1-2500	12.0	0.998
His	1-2500	9.3	0.998
Tyr	1-2500	13.6	0.996
Phe	1-2500	4.0	0.995
Trp	1-2500	20.5	0.996
Cit	1-2500	2.5	0.997
Kyn	1-2500	22.5	0.998

Table S4 Analytical method validation results of DSW as of recovery rate and repeatability

Analytes		spiked concentration /nmol/L	Intra-day		Inter-day	
			Accuracy, %	Precision, %	Accuracy, %	Precision, %
Gly	Null	25	100.1	0.8	100.5	1.8
		250	100.6	1.5	101.2	1.5
		1500	101.0	0.3	101.5	0.8
Ala	D	5	100.9	0.8	100.1	2.1
		50	100.9	0.8	101.1	1.0
		300	101.5	2.5	101.4	1.5
	L	25	99.6	3.1	100.6	1.9
		250	100.9	0.9	100.7	1.8
		1500	101.3	1.2	100.3	0.6
Ser	D	5	100.2	1.5	100.8	1.8
		50	100.4	0.5	100.8	2.4
		300	99.9	1.9	100.0	1.5
	L	25	100.8	0.8	100.8	1.2
		250	100.6	0.1	101.0	0.4
		1500	100.3	0.1	101.4	1.9
Thr	D	5	99.9	0.7	101.3	1.5
		50	99.8	1.4	100.3	0.5
		300	99.1	0.7	101.0	0.7
	L	25	100.6	1.9	99.5	1.9
		250	99.8	2.0	101.0	1.0
		1500	101.0	0.9	100.8	0.9
Pro	D	5	99.6	0.8	100.6	1.1
		50	100.0	3.1	101.5	1.0
		300	100.6	0.7	101.1	1.2
	L	25	101.9	1.1	102.8	1.3
		250	100.7	2.4	101.3	2.2
		1500	100.2	0.2	100.8	0.8
Val	D	5	100.8	1.2	102.2	2.1
		50	100.2	1.5	101.0	0.7
		300	101.1	1.3	100.9	1.1
	L	25	99.4	3.2	99.8	1.2
		250	100.2	0.1	101.2	1.9
		1500	100.5	0.2	99.9	0.9
Leu	D	5	101.8	3.8	102.2	2.6
		50	100.9	3.1	101.6	1.6
		300	100.2	1.1	99.5	1.0
	L	25	102.4	1.0	101.4	2.8
		250	100.9	3.1	99.2	0.6
		1500	100.2	1.1	100.5	0.6

Table S4 Analytical method validation results of DSW as of recovery rate and repeatability (Continues)

Analytes		spiked concentration /nmol/L	Intra-day		Inter-day	
			Accuracy, %	Precision, %	Accuracy, %	Precision, %
Ile	D	5	99.2	1.0	101.7	1.7
		50	101.1	0.8	99.0	0.6
		300	101.1	1.9	101.1	0.3
	L	25	100.7	1.4	101.9	1.1
		250	100.7	0.4	100.2	2.2
		1500	100.3	2.0	100.9	0.6
Met	D	5	99.7	0.9	103.8	1.4
		50	101.3	0.8	102.5	1.2
		300	100.4	0.9	101.9	1.5
	L	25	100.7	0.9	101.0	1.2
		250	101.5	0.8	100.7	0.7
		1500	99.0	0.4	101.5	0.4
Glu	D	5	100.9	0.5	100.0	2.5
		50	101.4	2.1	102.2	1.0
		300	100.6	2.7	101.1	1.0
	L	25	101.7	1.1	101.0	1.4
		250	102.6	1.2	101.3	1.0
		1500	100.0	0.5	100.9	0.5
Gln	D	5	100.9	2.4	100.8	1.4
		50	101.0	0.3	101.8	0.9
		300	100.7	0.7	100.4	1.5
	L	25	99.9	0.8	101.1	0.9
		250	101.0	0.1	101.3	1.1
		1500	101.0	0.1	101.9	1.9
Asp	D	5	100.7	2.4	103.8	1.4
		50	102.0	1.1	101.5	2.0
		300	100.7	1.0	101.2	1.2
	L	25	100.6	0.2	101.0	1.5
		250	100.8	0.1	101.4	0.5
		1500	100.8	0.3	99.9	0.6
Asn	D	5	100.1	0.5	100.6	1.7
		50	100.2	2.2	100.7	2.4
		300	100.1	0.5	100.9	2.5
	L	25	99.3	2.8	102.0	3.1
		250	100.9	1.2	100.9	1.4
		1500	99.0	0.9	100.5	1.1
Lys	D	5	98.3	3.5	103.0	4.5
		50	100.5	1.1	100.8	1.9
		300	100.5	2.8	101.7	2.0
	L	25	101.8	1.4	101.0	0.3
		250	101.7	0.8	102.6	1.0
		1500	101.0	0.3	100.8	0.6

Table S4 Analytical method validation results of DSW as of recovery rate and repeatability (Continues)

Analytes		spiked concentration /nmol/L	Intra-day		Inter-day	
			Accuracy, %	Precision, %	Accuracy, %	Precision, %
Arg	D	5	99.0	0.2	99.2	1.0
		50	100.9	0.7	101.1	0.6
		300	101.8	0.9	101.1	1.5
	L	25	100.5	0.3	99.6	0.9
		250	100.1	0.8	100.4	1.9
		1500	100.5	4.3	100.4	1.9
Cit	D	5	98.1	0.5	100.8	1.4
		50	96.4	2.3	101.3	1.8
		300	101.2	1.5	100.4	1.7
	L	25	97.5	1	96.8	1.0
		250	104	0.7	102.6	0.6
		1500	99.2	0.7	100.0	0.7
His	D	5	100.6	1.3	101.3	2.7
		50	98.5	3.1	101.5	1.7
		300	101.1	2.2	102.8	1.0
	L	25	100.0	0.1	102.2	1.5
		250	99.6	0.4	100.4	0.8
		1500	101.3	0.8	100.7	1.5
Tyr	D	5	100.2	1.7	100.8	1.9
		50	100.9	1.5	101.0	0.9
		300	101.5	1.3	102.1	1.4
	L	25	99.5	2.9	100.6	0.4
		250	100.9	0.7	100.3	0.1
		1500	100.5	0.2	100.4	1.2
Phe	D	5	99.2	0.1	99.9	2.2
		50	99.5	1.2	102.0	1.1
		300	99.6	1.1	100.2	0.9
	L	25	99.1	1.1	100.0	1.9
		250	101.0	0.6	102.0	0.9
		1500	100.6	0.2	100.2	0.7
Trp	D	5	100.8	2.0	101.4	0.8
		50	99.1	5.0	101.8	2.4
		300	100.2	1.7	101.3	1.3
	L	25	99.7	0.7	100.2	1.1
		250	101.6	2.0	102.8	1.2
		1500	100.4	1.3	100.6	1.6
Kyn	D	5	96.5	2.4	96.1	1.7
		50	102.1	0.8	102.2	1.2
		300	96.8	1.4	98.7	3.8
	L	25	95.5	2.2	103.2	1.1
		250	105.7	3.7	99.2	1.7
		1500	99.4	0.7	99.7	0.8

Table S5. Recovery rate evaluation with chemical tagging reaction and matrix effect from DSW.

Analytes (Abbreviation)		Reaction recovery rate Average (%) / RSD (%)	Matrix effect Average / RSD (%)
Gly	Null	75.5 / 6.0	1.115 / 6.2
Ala	D	63.0 / 8.9	1.082 / 5.8
	L	71.7 / 7.4	0.982 / 9.5
Ser	D	61.3 / 11.4	0.915 / 2.3
	L	60.8 / 8.9	1.123 / 6.8
Pro	D	66.5 / 5.2	0.855 / 5.4
	L	63.9 / 3.1	1.012 / 6.8
Val	D	65.7 / 11.8	0.899 / 7.7
	L	71.1 / 5.9	0.956 / 9.5
Thr	D	68.2 / 6.0	1.071 / 6.3
	L	70.6 / 6.7	1.035 / 5.4
Ile	D	71.7 / 12.9	0.956 / 8.2
	L	64.0 / 5.9	0.895 / 10.7
Leu	D	61.8 / 6.5	1.113 / 10.1
	L	61.6 / 8.2	1.042 / 9.2
Asn	D	63.4 / 10.9	0.877 / 11.5
	L	63.6 / 9.6	1.051 / 8.5
Asp	D	65.3 / 7.7	1.126 / 7.6
	L	63.8 / 9.0	1.015 / 5.8
Gln	D	68.7 / 12.7	0.936 / 6.7
	L	62.5 / 12.6	0.924 / 7.5
Lys	D	58.5 / 9.1	0.886 / 11.5
	L	59.3 / 5.8	0.915 / 8.5
Glu	D	70.4 / 11.1	1.056 / 3.8
	L	66.7 / 7.1	1.115 / 4.8
Met	D	67.7 / 6.3	0.987 / 8.0
	L	66.8 / 10.4	1.025 / 9.6
His	D	65.6 / 7.6	0.897 / 7.5
	L	70.9 / 4.2	1.002 / 9.5
Phe	D	69.3 / 8.0	1.065 / 2.8
	L	64.9 / 10.9	1.008 / 5.3

Arg	D	64.4 / 11.1	1.025 / 5.5
	L	60.4 / 7.1	0.985 / 3.4
Cit	D	73.7 / 4.3	1.098 / 9.6
	L	77.2 / 9.1	0.967 / 5.6
Tyr	D	66.0 / 10.1	0.997 / 6.3
	L	61.4 / 5.4	0.926 / 7.9
Trp	D	69.5 / 7.5	1.022 / 9.9
	L	68.7 / 7.4	0.945 / 8.2
Kyn	D	75.4 / 2.3	0.994 / 7.1
	L	70.9 / 3.5	1.014 / 3.0

Each test was conducted for N=6.

Table S6. The result of log-rank test of *C.elegans* life span assay.

Log-rank test	PC	Met	2.5%DSW	10%DSW
Control				
PC	1.8E-09			
2.5%DSW	6.5E-09	1		
10%DSW	0.000075	0.29	0.81	

Table S7. The comparison of the performance of the enantio-chemical tags.

Reagent name	Reaction condition	Reaction time needed to plateau	Measurement time	Simultaneous range of DL-AAs	Sensitivity based on LOQ of the method
L-FDLA	55°C	40 min	45 min	14 DL-AAs	100 ~ 1000 pmol/L
(*S*)-NIFE	R.T.	20 min	25 min	19 DL-AAs	1 nmol/L
OPA/IBLC	R.T.	2 min (stable for at least 40 min)	34.5 min	16 DL-AAs	9.21 ~ 206 pmol/L
DMT-(*S*)-Pro-OSu	R.T.	40 min	45 min	18 DL-AAs	18.4 pmol/L ~ 1.19 nmol/L
NCS-OTPP	60°C	60 min	25 min	2 DL-AAs	7.92 ~ 23.8 nmol/L
DIPP-L-Ala-NHS	R.T.	15 min	41 min	20 DL-AAs	40 pmol/L ~ 200 nmol/L
D-BPCI	4°C	Over night	24 min	13 DL-AAs	2.50 ~ 500 nmol/L
DATAN	75°C	120 min	75 min	20 DL-AAs or more	3.33 ~ 179 nmol/L
(*S*)-COXA-Osu	40°C	90 min	60 min	20 DL-AAs or more	9.22 ~ 346 nmol/L
CMT-D-Leu*	80°C	10 min	17 min	20 DL-AAs	0.33 ~ 54.9 pmol/L

*: The novel tag developed in this study. R.T.: Room temperature.
The references were obtained from the review article; Lella, C.; Nestor, L.; De Bundel, D.; Vander Heyden, Y.; Van Eeckhaut, A. Targeted Chiral Metabolomics of D-Amino Acids: Their Emerging Role as Potential Biomarkers in Neurological Diseases with a Focus on Their Liquid Chromatography–Mass Spectrometry Analysis upon Chiral Derivatization. *Int. J. Mol. Sci.* **2024**, 25, 12410. https://doi.org/10.3390/ijms252212410
